# A Rare Presentation of Pancreatic Lymphoepithelial Cyst: A Case Report and Review

**DOI:** 10.1155/2020/4590758

**Published:** 2020-02-13

**Authors:** Grace E. Kim, Fariha Ramay, Stephanie Richards, Peter E. Darwin

**Affiliations:** ^1^University of Maryland Medical Center, Department of Internal Medicine, Baltimore, Maryland, USA; ^2^University of Maryland School of Medicine, Division of Gastroenterology and Hepatology, Baltimore, Maryland, USA; ^3^University of Maryland Medical Center, Department of Pathology, Baltimore, Maryland, USA

## Abstract

Pancreatic lymphoepithelial cyst (LEC) is a rare, benign collection of keratinizing squamous epithelial cells encapsulated by lymphoid tissue. Because of its limited data and nonspecific features that can mimic malignant lesions, LECs can lead to unnecessary operations. A 62-year-old male with a known pancreatic mass presented with abdominal pain. CT scan showed an increased mass in the pancreatic head, and endoscopic ultrasound-guided fine needle aspiration (EUS-FNA) revealed “rare fragments of benign-appearing squamous epithelium in a background of keratin debris, cyst contents, and scattered lymphocytes,” consistent with a lymphoepithelial cyst. Pancreatic LEC is an extremely rare lesion that comprises of only 0.5% of all pancreatic cysts. EUS-FNA has become the mainstay for diagnosing pancreatic LECs. Given the slow growing and benign nature, conservative management and observation is adequate for pancreatic LECs with excellent long-term outcome. With increasing number of imaging ordered by clinicians, it is anticipated that there will be a greater number of incidental pancreatic LECs detected. Thus, EUS-FNA should be utilized more frequently to help distinguish benign pancreatic LECs from premalignant or malignant lesions to avoid surgery.

## 1. Introduction

Pancreatic lymphoepithelial cyst (LEC) is a rare, benign collection of keratinizing squamous epithelial cells encapsulated by lymphoid tissue [1, 2]. About 200 cases of LEC have been described in English literature since the 1980s [1, 3, 4], with the first case described in German literature [5]. Because of its limited data and nonspecific features that are usually described only in case series, the pathogenesis, clinical features, and diagnosis of LEC are not well understood. Pancreatic LECs may also have elevated tumor markers such as CEA and CA 19-9 levels [3, 6–10] with nonspecific features that can mimic malignant lesions, leading to unnecessary operations. Here, we review a rare case of pancreatic LEC with a unique cytopathological image.

## 2. Case Report

**Figure 1:**
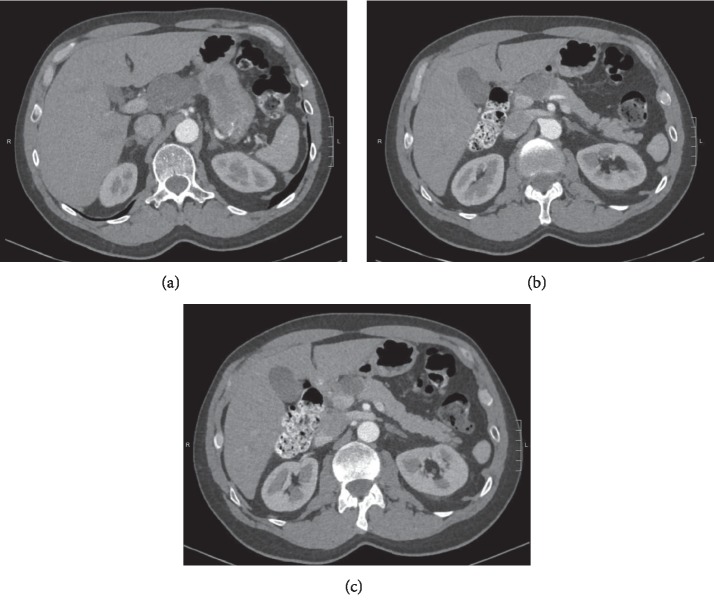
Series of images (proximal to distal) from abdominal CT showing a 36 × 52 mm mass in the pancreatic head with no pancreatic ductal dilation.

**Figure 2:**
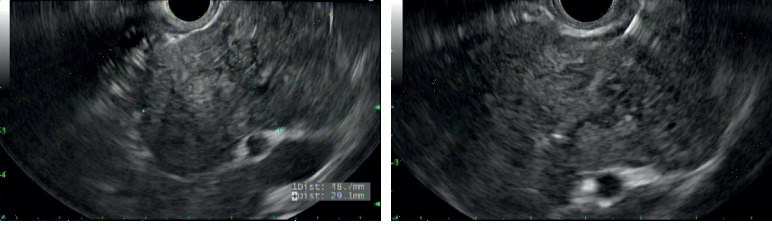
EUS showing a 49 × 29 mm heterogenous hypoechoic mass by the pancreatic neck, again without any pancreatic ductal dilation.

**Figure 3:**
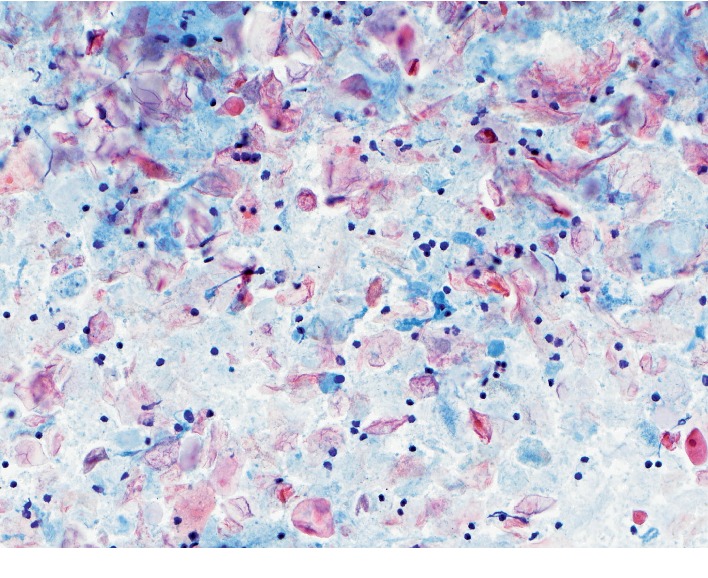
Cytology smear of cyst aspirate showing numerous anucleate squamous cells and keratin debris with scattered background lymphocytes (Papanicolaou stain, original magnification 20x).

A 62-year-old male with a known pancreatic mass presented with a nonspecific abdominal pain. He had an abdominal ultrasound performed about 7 years before that showed a suspected 4.7 × 3.4 × 5.2 cm mass in the region of the pancreatic head. This was not further addressed, and the patient was not referred. He then presented to the ED 4 years later for chest and back pain for which he received a CTA aorta showing a 4.5 × 2.9 × 3.8 cm hypoenhancing mass by the superior pancreatic neck with no pancreatic ductal dilation or atrophy. This was followed up by an MRI of abdomen that year that showed a 2.6 × 3.8 cm heterogenous nodule in the pancreatic head, high signal on T2, and hypointensity on T1 weighted imaging, without any ductal dilation. Again, he was lost to follow-up but represented with abdominal pain 3 years later, prompting a right upper quadrant ultrasound that showed a suspected lesion near the head of the pancreas, measuring 4.7 × 3.4 × 5.2 cm. Subsequent CT of the abdomen showed a 3.6 × 5.2 cm mass in the pancreatic head with no pancreatic ductal dilation or retroperitoneal lymphadenopathy (Figure 1). Given the interval increase in size in conjunction with his abdominal pain, patient underwent EGD with EUS which showed a well-defined, avascular 49 × 29 mm heterogenous hypoechoic mass by the pancreatic neck without any pancreatic ductal dilation or abnormalities (Figure 2). Subsequent EUS-guided fine needle aspiration (FNA) with a 25-gauge needle revealed “rare fragments of benign-appearing squamous epithelium in a background of keratin debris, cyst contents, and scattered lymphocytes,” consistent with a lymphoepithelial cyst (Figure 3). The patient opted for a conservative management. Subsequent follow-up of abdominal ultrasound 3 months later showed a hypoechoic mass in the pancreatic head measuring 4.7 × 3.5 × 5.4 cm, stable when compared with the prior study. The patient had intermittent diarrhea with abdominal pain in the lower quadrants during the follow-up appointment, and further evaluation is ongoing at this time with a repeat MRI of the abdomen pending.

## 3. Discussion

Pancreatic LEC is an extremely rare lesion that comprises of only 0.5% of all pancreatic cysts [11]. They are usually around 6 cm in size although it can range from 1 to 15 cm [11–13]. Its pathogenesis is unclear, but it has been proposed that pancreatic LEC could be an ectopic pancreatic tissue in a peripancreatic lymph node, an ectopic accessory spleen, or a remnant of a bronchial cleft tissue [2, 12].

Pancreatic LEC predominantly occurs in males aged 50 to 60 years [3, 6, 7, 12, 14, 15], which is on par with when the patient in our case initially presented. It is commonly an incidental finding, albeit patients may present with nonspecific symptoms such as abdominal pain and nausea [9, 12, 16, 17]. Majority of the LECs are well-defined, round, anechoic, or hypoechoic complex cystic lesions with enhancing septa or rim, uniformly distributed in an exophytic location around the head, body, and tail of the pancreas [3, 7, 15, 18, 19]. However, these features can overlap with other types of pancreatic lesions such as intraductal papillary mucosal neoplasms or mucinous cystic neoplasms which have malignant potential, leading to unnecessary surgical intervention. Mege et al. in his review of 117 patients with pancreatic LECs stated that only 22% of patients had an accurate preoperative diagnosis of pancreatic LEC [3]. CT and MRI can aid in further distinguishing pancreatic LEC from other pancreatic lesions; for example, studies report pancreatic LEC on MRI show low intensity on T1 imaging, whereas it has high intensity of T2-weighted just like the MRI findings of our patient depicted in the case. However, imaging alone is not reliable as dermoid and epidermoid cysts can also have similar findings on imaging, and recent studies have shown that increasing number of cases had high diagnostic accuracy with EUS-FNA preoperatively [16, 20–23].

Hence cytopathological evaluation through EUS-FNA has become the mainstay to increase accuracy of diagnosis for pancreatic LECs. LEC tends to have common distinct features comprised of an outer layer of lymphoid tissue and an inner layer of mature, nucleated squamous epithelium without atypia [2, 3, 6, 15, 16, 24, 25]. Most specific cytopathological features include cholesterol crystals, keratin, and squamous cells, or fragments [26]. The cyst lumen comprises keratinized debris giving cheese-like, caseous appearance [2, 25, 27]. These features are similar to those of dermoid and epidermoid cysts, but LEC is more common in males whereas the latter two do not have gender preference [16]. Moreover, while LECs have lymphoid follicles, dermoid cysts have mucinous cells with sebaceous respiratory tissue, and epidermoid cysts have splenic tissues [17, 28, 29]. Since the patient in our case lacked the latter two findings while having lymphocytes present on cytopathology, this further confirms that the patient had a pancreatic LEC. Cytopathologic characteristics of pancreatic LEC can also mimic malignant lesions such as pancreatic adenocarcinoma, but LECs lack cytological atypia [21, 25]. Currently, there are a limited data in the U.S. regarding the sensitivity and specificity of EUS in diagnosing LECs given broad differential diagnoses of pancreatic cysts. In one recent retrospective study, only 10 out of 29 patients with LEC had the diagnosis exclusively based on imaging (EUS and CT) [15]. Thus, tissue acquisition via EUS-FNA can be very useful in distinguishing specific types of pancreatic lesions and should be utilized to work up suspected LEC [30]. Nonetheless, even with EUS-FNA, the diagnosis of LEC can be challenging due to insufficient sample size and overlapping cytopathological features, with one study reporting that FNA may yield 38% of specific features of LEC mentioned above [26]. EUS-FNA with or without tumor marker assay such as CEA and CA 19-9 has been proposed as a diagnostic tool for pancreatic LECs [10, 19, 24, 31]. Our patient has not had CEA or CA 19-9 checked, but the FNA sample was sufficient to make the diagnosis of FNA.

Given the slow growing and benign nature, conservative management and observation is adequate for pancreatic LECs with excellent long-term outcome [2, 3, 7, 10, 19, 25, 30]. They can also be treated with simple excision of the cyst or distal pancreatectomy for symptomatic patients or if malignancy cannot be excluded [2, 10, 19, 30]. In our case, the patient was agreeable to first undergoing a trial of conservative management with symptomatic control prior to considering surgical intervention for his cyst.

While there is a general consensus of how pancreatic LECs present under various imaging modalities, pancreatic LECs are still a rare entity and can portray different features in EUS depending on the content of mucin, keratin, and lymphoid tissue in the cyst [6]. With increasing number of imaging ordered by clinicians, it is anticipated that there will be a greater number of incidental pancreatic LECs detected, which in turn can further increase the number of unnecessary procedures. Demographically, these middle-aged or elderly males may have multiple comorbidities and could be poor surgical candidates; thus, EUS-FNA should be utilized more frequently to help distinguish these benign pancreatic LECs from premalignant or malignant lesions to avoid surgery. It is important for clinicians to become more familiarized with the cytopathological characteristics of pancreatic LECs, and this case highlights the importance of EUS-FNA in accurately diagnosing a rare pancreatic LEC.
